# Identification and Functional Clustering of Genes Regulating Muscle Protein Degradation from amongst the Known *C. elegans* Muscle Mutants

**DOI:** 10.1371/journal.pone.0024686

**Published:** 2011-09-27

**Authors:** Freya Shephard, Ademola A. Adenle, Lewis A. Jacobson, Nathaniel J. Szewczyk

**Affiliations:** 1 School of Graduate Entry Medicine and Health, University of Nottingham, Royal Derby Hospital, Derby, United Kingdom; 2 Department of Biological Sciences, University of Pittsburgh, Pittsburgh, Pennsylvania, United States of America; Inserm U869, France

## Abstract

Loss of muscle mass via protein degradation is an important clinical problem but we know little of how muscle protein degradation is regulated genetically. To gain insight our labs developed *C. elegans* into a model for understanding the regulation of muscle protein degradation. Past studies uncovered novel functional roles for genes affecting muscle and/or involved in signalling in other cells or tissues. Here we examine most of the genes previously identified as the sites of mutations affecting muscle for novel roles in regulating degradation. We evaluate genomic (RNAi knockdown) approaches and combine them with our established genetic (mutant) and pharmacologic (drugs) approaches to examine these 159 genes. We find that RNAi usually recapitulates both organismal and sub-cellular mutant phenotypes but RNAi, unlike mutants, can frequently be used acutely to study gene function solely in differentiated muscle. In the majority of cases where RNAi does not produce organismal level phenotypes, sub-cellular defects can be detected; disrupted proteostasis is most commonly observed. We identify 48 genes in which mutation or RNAi knockdown causes excessive protein degradation; myofibrillar and/or mitochondrial morphologies are also disrupted in 19 of these 48 cases. These 48 genes appear to act via at least three sub-networks to control bulk degradation of protein in muscle cytosol. Attachment to the extracellular matrix regulates degradation via unidentified proteases and affects myofibrillar and mitochondrial morphology. Growth factor imbalance and calcium overload promote lysosome based degradation whereas calcium deficit promotes proteasome based degradation, in both cases myofibrillar and mitochondrial morphologies are largely unaffected. Our results provide a framework for effectively using RNAi to identify and functionally cluster novel regulators of degradation. This clustering allows prioritization of candidate genes/pathways for future mechanistic studies.

## Introduction

Muscle mass is maintained by the balance of protein synthesis and degradation [1], termed proteostasis [2]. Global proteostasis has been postulated to create molecular robustness that underlies how genetic diversity can be maintained in the absence of strong selective pressures [3]. In muscle, proteostasis is key to maintaining contractile ability and therefore locomotion. Additionally, because muscle is the body's major reservoir of protein for catabolism in time of need, the ability to adjust to a different proteostatic state is important to maintenance of overall physiological homeostasis in the organism. Numerous clinical conditions are associated with loss of muscle proteostasis. These are not limited to rare disorders such as the muscular dystrophies, but include conditions associated with major healthcare expenditure such as cancer (cachexia), aging (sarcopenia), heart failure, and diabetes [4], [5]. While much work has been devoted to the problem, it is largely unknown how extramuscular signals regulate the key proteolytic systems within muscle. For example, in humans more than a dozen extramuscular signals are associated with muscle atrophy [1] and four key proteolytic systems exist [6], yet we know very little of how these signals regulate the proteases, and in many cases we are uncertain which proteases they regulate.

To address these gaps in knowledge, the nematode *C. elegans*, a convenient organism for systems biology, has been developed as an experimental model for studying the intramuscular signals that regulate muscle protein degradation. It has previously been shown that proteasome based degradation is opposed by signal from motor neurons [7], as is suspected for human muscle. Additionally, lysosome based degradation is opposed by a balance between pro-degradation signal via FGFR-Ras-Raf-MAPK [8], [9] and anti-degradation signal via IGFR-PI3K-Akt-Raf [10]. Both signals are thought to be important in mammalian muscle, although which protease(s) they regulate remains an open question. The work to develop *C. elegans* into a model for understanding the regulation of muscle protein degradation has taken more than fifteen years, and has uncovered novel functional roles for genes previously identified as the sites of mutations affecting muscle and/or as involved in signalling in other cells or tissues.

Much work on *C. elegans* focuses on the genetic regulation of behaviour [11]; thus, we currently know mutations in roughly 234 genes that affect muscle development and/or physiology in the form of altered locomotion (*unc*) [12], egg laying (*egl*) [13], feeding (*eat*) [14], defecation (*exp*, *pbo*, *dec*, *aex*) [15], or attachment (*mua*, *mup*, *rol*) [16]. We anticipated that these classes of previously identified genes would be enriched for negative regulators of muscle protein degradation. Testing this prediction by traditional approaches would have required several hundred genetic constructions to place the appropriate transgenic reporter(s) of proteostasis together with the mutation under study. We therefore chose to use RNAi to interrogate the roles of these genes in maintaining proteostasis. Here we use a combination of mutants and RNA interference (RNAi) to analyse a large subset of previously identified genes regulating muscle development and/or physiology, with the primary aim of identifying genes regulating muscle protein degradation. We selected 159 of the 234 genes that affect muscle development and/or physiology for analysis. These 159 genes were selected because RNAi clones against these genes were commercially available and past results using these clones were published. The selection of this set of genes for analysis also enabled our secondary aims: i) to conduct an assessment of the accuracy and reproducibility of RNAi; ii) to conduct a comparative assessment of RNAi and traditional genetic approaches for identifying and studying genes regulating muscle protein degradation. Of the 159 genes studied, 48 were identified as novel regulators of muscle protein degradation. This finding confirmed the prediction that the classes of genes studied would be enriched for negative regulators of degradation. In addition to identifying novel genes/pathways for future mechanistic work, the comparative studies allowed us to develop a strategy for integrating new RNAi experiments with the past genetic and pharmacologic approaches to this problem. Utilizing this strategy we have clustered the 48 genes into 3 functional groups. Two of these groups associate new genes with known signalling networks while the third group appears to represent at least one new signalling network. Thus, our strategy allows newly identified genes to be prioritized for future studies of either novel regulators of degradation or modifiers of known signalling networks.

## Results

### RNAi feeding produces accurate functional data that are largely reproducible

Compared to traditional genetic analysis [12], the use of RNAi is relatively recent [17]. We were therefore concerned about reproducibility and reliability of results obtained by RNAi [18]. From the 234 genes that affect muscle development and/or physiology we identified 159 genes for which RNAi feeding vectors were used in published large scale RNAi based screens [19], [20], [21]. We used these vectors and found false positive and negative rates consistent with past suggestions for this RNAi technique [20]; <1% and 39% respectively, based upon consistency of behavioural and/or developmental phenotype observed by RNAi and the known mutant phenotype (Table S1). We next compared our results for behavioural and/or developmental defects to those obtained by others using the same vectors (Table S1). This analysis revealed that the majority of our observations were consistent with past reports (Fig. S1). However, a substantial number of genes for which RNAi had not previously been reported to produce a behavioural and/or developmental phenotype did produce a phenotype in our experiments that was consistent with the known mutant phenotype. Together, these results suggest that use of RNAi feeding vectors that are known to produce a phenotype, along with appropriate replicates and controls, can produce a low false negative rate (3–5% in this study; Fig. S1). Given that 6% of our results recapitulate a mutant phenotype but a different developmental/behavioural phenotype was previously reported by RNAi in a wild-type genetic background (Fig. S1), we suspect that 10% (false positives [<1%]+false negatives [3%]+phenotypic divergence [6%]) is a realistic estimate for the lower limit of observed experimental discrepancies of bacterial feeding vector RNAi experiments in *C. elegans*. We will further address the issue of incomplete knockdown below.

### Identification of genes affecting sub-cellular muscle compartments

**Figure 1:**
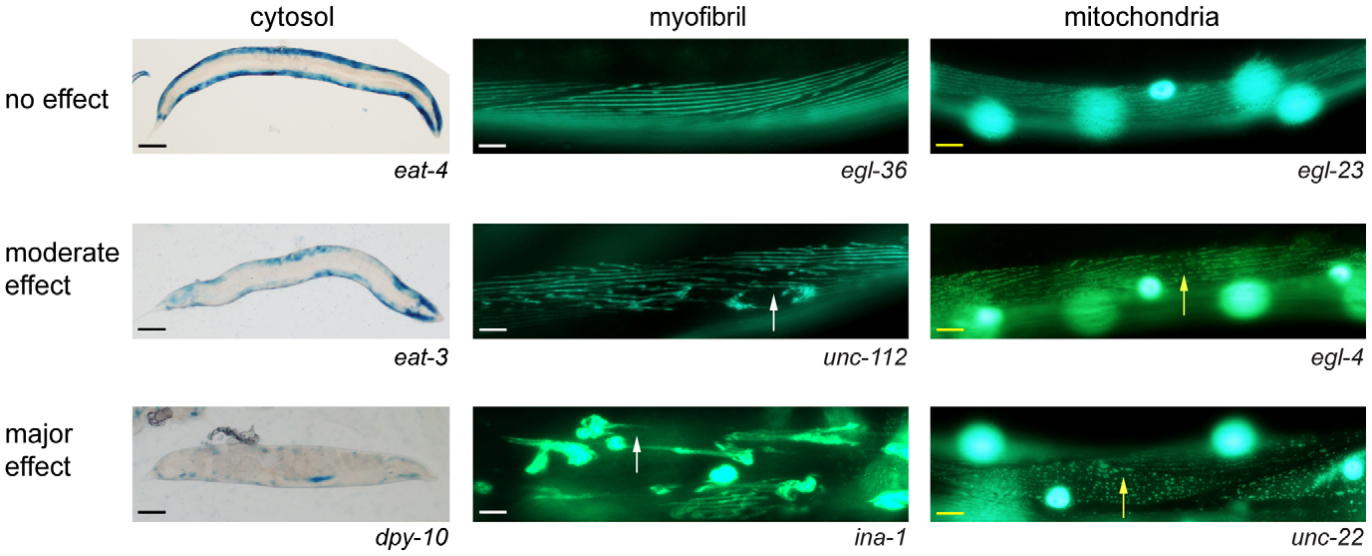
Sub-muscular phenotypes produced by *C. elegans* RNAi feeding vectors. Examples of sub-cellular phenotypes scored. Cytosolic protein content (left), myofibrillar morphology (middle), and mitochondrial morphology (right) were scored as normal (top) or abnormal (middle and bottom); abnormalities were only scored if they were considered moderate or major (see Materials and methods for additional details). Separate scale bars are used for each sub-cellular phenotype (cytosol: black bar, 100 µm; myofibril: white bar, 10 µm; mitochondria: yellow bar, 10 µm). Arrows indicate gaps in the myofibrils (white) and mitochondrial networks (yellow). The large, circular, over exposed regions in the mitochondrial images are GFP labelled nuclei, which are all normal. The large, rounded, over exposed regions in the major effect myofibril image are aggregates of myosin and are not normal.

**Figure 2:**
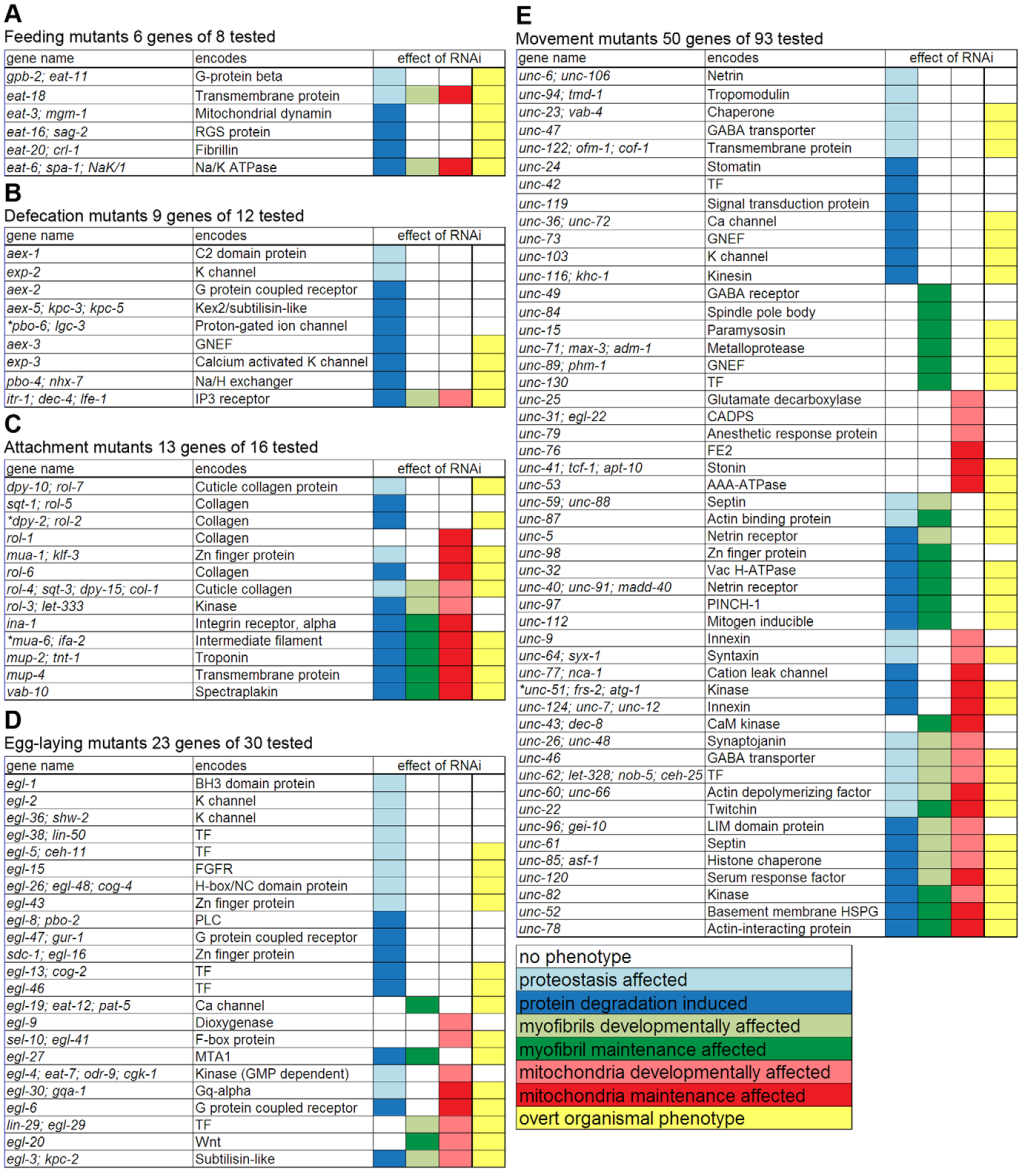
Genes that affect muscle protein synthesis, degradation and/or dystrophy. Effect of RNAi on sub-cellular compartments of muscle are displayed with data colour coded to inset legend. Data are listed for each gene named to the left. Genes are broken into classes: A) *eat* mutants; B) *aex*, *exp*, *pbo*, *dec* mutants; C) *mua*, *mup*, *rol* mutants; D) *egl* mutants; E) *unc* mutants. Genes within each class are clustered by which compartment(s) are effected with the root order: cytosol, myofibril, and mitochondria. Priority in rooting is given to developmental effect and the root ordering is reflected in the order of colours in the inset legend. For comparison, presence (yellow) of organismal level phenotypes is indicated to the right and given last priority in rooting. In all cases white indicates lack of effect. Examples of sub-cellular phenotypes that were scored are provided in Figure 1 and genes from the same classes that did not display sub-cellular defects are shown in Figure S2. Four genes (*pbo-6*, *dpy-2*, *mua-6*, *unc-51*) were identified as potential false positives for RNAi inducing protein degradation; these genes are indicated with asterisks as RNAi against them may produce variable results and/or be false positives for inducing degradation.

Past work using transgene-coded reporter proteins in *C. elegans* has shown that in some conditions that promote bulk protein degradation in muscle cytosol, nuclear and myofibrillar proteins are largely unaffected [7], [8], [9], [10], [22], [23], while mitochondria are affected in some cases but not others (LAJ & NJS unpublished observations). This is similar to the situation in humans, where distinct regulation of sub-cellular compartments in muscle is suspected [24]. Therefore, in addition to cytosolic protein content, we assessed myofibrillar and mitochondrial structure in response to chronic (inter-generation) RNAi treatment throughout development, and followed up positive findings with additional acute (intra-generation) RNAi treatments in fully developed adults (Fig. 1, Fig. 2, Fig. S2). An acute (intra-generation) effect of RNAi implies that the gene product is required in adults to prevent these abnormalities. Note that because the *lacZ* transgene used to study cytosolic protein is only expressed until adulthood and the product is not degraded for at least the next 72 hours [7], [8], [9], [10], [22], [23], changes in response to chronic RNAi treatment show a defect in proteostasis without distinguishing synthesis from degradation, whereas changes in response to acute RNAi treatment must be the result of degradation alone. Using this approach we identified 101 genes as affecting sub-cellular muscle compartments (Fig. 2).

### Cytosolic proteostasis is the most commonly affected sub-cellular process

**Figure 3:**
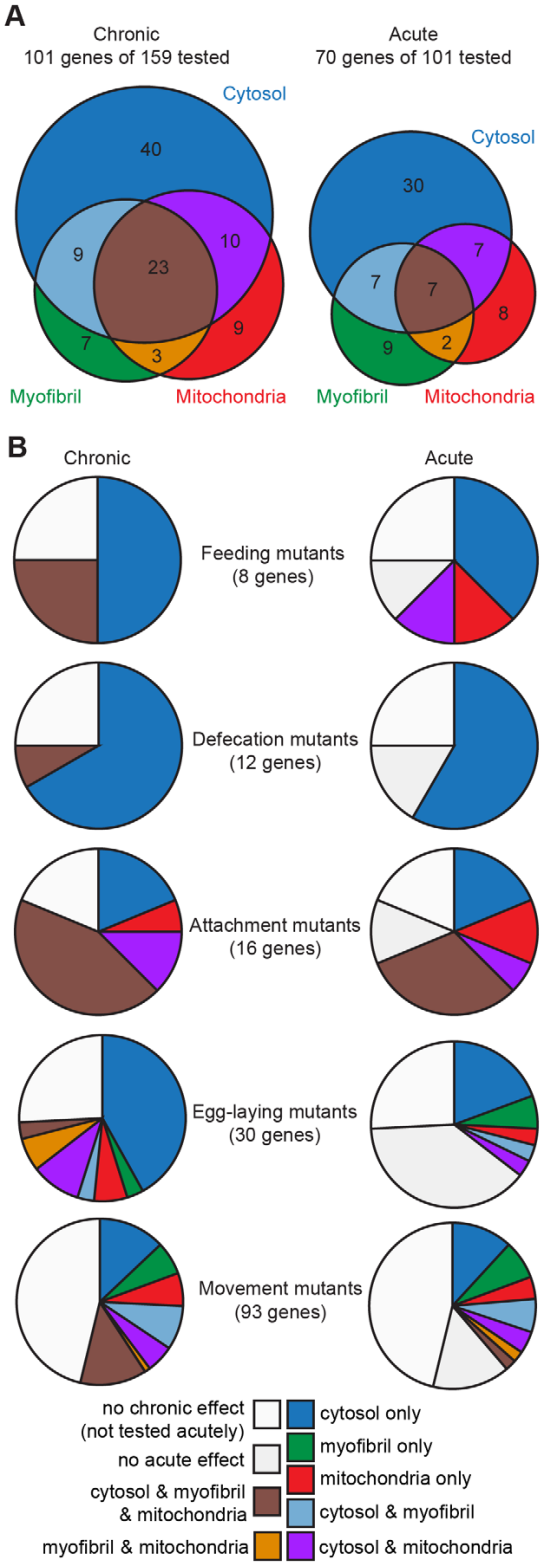
Distribution of sub-cellular compartments affected. A) Global analysis of data presented in Figure 2. Number of genes whose products are required to maintain proteostasis (Cytosol), myofibrillar morphology (Myofibril), and/or mitochondrial morphology (Mitochondria) during development, as assayed by the effect of chronic, inter-generational, RNAi treatment (Chronic, left). Number of genes whose knockdown affects protein degradation (Cytosol), myofibrillar maintenance (Myofibril), and/or mitochondrial morphology (Mitochondria) in fully developed adult muscles, as assayed by the effect of RNAi in adults (Acute, right). Note that acute studies were only conducted on those genes for which a chronic effect was observed. B) Gene class analysis of data presented in Figure 2. Sub-cellular compartments that display defects in response to RNAi are displayed as number of genes affecting each compartment. Graphs are displayed by gene class (labels in middle, total number of genes in class in parenthesis) with separate graphs for defects observed in response to chronic RNAi treatment (Chronic, left) or in fully developed adults (Acute, right). The gene classes that correspond to the labels are as follows: Feeding mutants, eat; Defecation mutants, *aex*, *exp*, *pbo*, *dec*; Attachment mutants, *mua*, *mup*, *rol*; Egg laying mutants, *egl*; Movement mutants, *unc*. An inset legend identifies the affected compartments. The colour codes are the same for A and B. The root ordering from Figure 2 is used in Figure 3B with effected compartments starting at the 12 o'clock position and moving clockwise (e.g. cytosol only, myofibril only, etc.); the root ordering and clockwise rotation are reflected in the order of the inset legend. Note that if RNAi against a gene did not produce an effect chronically, RNAi against the gene was not tested acutely.

Both chronic and acute RNAi treatments result in muscle compartment specific defects (Fig. 2, Fig. 3A). Strikingly, the muscle cytosol is the compartment most likely to show an effect in response to RNAi knockdown of one of these genes known to affect muscle physiology and/or development (82/101). This suggests that the biochemical prioritization in muscle treats the soluble cytosolic proteins required to power contractions as more dispensable for maintenance of organismal homeostasis than the proteins that make up the contractile apparatus. This fits with the hypothesis that proteostasis serves to provide a buffer against individual genetic disruptions [3].

### The majority of genes that affect sub-cellular muscle compartments during development continue to do so in fully differentiated muscle

As shown in Figure 3A, 70/101 genes identified as regulating muscle development also appear to have a role in maintaining terminally differentiated muscle (e.g. negatively regulating cytosolic degradation, myofibril disassembly, mitochondrial fragmentation) as evidenced by muscle defects in response to acute RNAi treatment. Intriguingly, more genes (23) are required for the proper development of all three compartments than appear to be important for the maintenance of all three compartments in terminally differentiated muscle (7 genes). This may suggest that, in *C. elegans*, more genes have been subject to selection for proper development than for proper maintenance of adult muscle and/or that it is easier to genetically perturb muscle during development than in adulthood. This is in apparent contrast to the function of insulin-like signalling which is important for metabolic capacity both during development and in adults [25].

### RNAi feeding produces sub-cellular phenotypes even in the absence of whole organism behavioural or developmental phenotypes

Large-scale RNAi screens and gene knockout studies have reported that disruption of many genes produces no effects visible at the level of the whole organism. It is therefore important to ask whether knockdown of many individual genes causes no overt phenotype due to functional redundancy or compensation, or if the phenotype resulting from knockdown is often subtle enough to escape detection [3]. Since the genes in the set we have knocked down are known to have visible behavioural and/or developmental defects when mutated, it seemed possible that the RNAi-treated animals that did not display overt phenotypes might simply have suffered incomplete knockdown, generating potential false negatives. In 58/90 cases we, like others, found no gross phenotypic effect in response to RNAi (Fig. S1). Gratifyingly, when we examined sub-muscular defects and combined this data with our data on behavioural/developmental defects (Fig. 2) we found that the majority of RNAi treatments that did not induce behavioural or developmental phenotypes did, in fact, induce sub-cellular defects (Fig. S1). Thus, our results appear to support the notion that many RNAi treatments produce phenotypes that are subtle enough to escape detection. As these sub-cellular defects result from gene knockdown, the lack of an overt phenotype may be due to the quantitative extent of knockdown. The ability to resist the negative consequences of decreases in gene expression would appear to support the notion that biological systems are robust at the organismal level. For example, individual genes may often be important at the molecular level, but control only small changes in overall fitness under non-selective conditions [3], [26].

### Behavioural phenotypes are associated with specific patterns of sub-cellular defects

The set of genes known to affect muscle physiology was originally identified based upon mutational effects on several different behavioural phenotypes. Thus, we analysed the frequency of compartment specific defects associated with each gene class. As shown in Figure 3B, *unc* genes (associated with movement defects) were less likely than the other gene classes to be associated with defects in sub-cellular muscle compartments, perhaps because many *unc* genes act principally in the nervous system and were thus unaffected by RNAi in a wild-type strain. Expression exclusively in neurons may account for 89% of *unc* genes that gave no effect when knocked down. It is also true that 74% of *unc* genes that did show sub-cellular muscle defects upon RNAi knockdown are expressed in neurons based on expression data in WormBase [27], but these genes may also be expressed in muscle (about half are already reported to be). Notably, the attachment and *unc* genes showed a different distribution of sub-cellular compartment defects than the other gene classes. The attachment genes encode products that not only anchor the contractile apparatus, but also provide structural interaction and communication with immediately adjacent cells, including other muscle cells. Knockdowns of attachment genes were more likely to show defects in all three compartments assayed, whereas the *unc* genes were more likely to show diverse compartment specific and multi-compartment defects. That is, disruption of muscle attachment is less well tolerated by various sub-cellular processes than is disruption of contraction. Consistent with this, acute RNAi knockdown of attachment genes in adults is most likely to give an effect in fully developed muscle and to disrupt the same compartments disrupted during development. This latter observation suggests that muscle cell attachment complexes are dynamic structures even in fully differentiated muscle, and that failure to maintain them has catastrophic consequences for muscle. Conversely, our observations with the *unc* genes suggest that while it may be easy to perturb contraction, there is robust protection against catastrophic structural problems in muscle.

### Integrating RNAi knockdown and mutational analysis

In order to assess how best to integrate RNAi experiments with our existing methods, we directly compared the use of RNAi to a traditional genetic approach. For the comparative analysis, we selected all muscle mutant genes that had previously been placed together with transgenic reporters of proteostasis as part of past and on-going work, and another group of genes for which dominant mutant alleles existed [28]. Since RNAi acts by reducing the amount of wild-type gene product rather than altering the function of the gene product, we anticipated that dominant mutant alleles (which might produce hyperfunctional gene products) would yield opposite effects. However, this was true for only 8 of the 14 dominant mutant alleles examined by RNAi (Table 1, Table S2). Of the 6 alleles that gave the same phenotypic results as RNAi, one has been described as a loss of function allele (*egl-30*) and one as a dominant negative allele (*unc-27*), so that similar results of mutation and RNAi knockdown are expected. Of the remaining four genes where neither gain-of-function mutations nor RNAi produced overt effects on muscle proteostasis, in two instances (*egl-19* and *unc-58*) reduction-of-function alleles also did not grossly perturb proteostasis. Combining the dominant alleles with the broader set of muscle mutants (Table 1, Table S2), we find that in the majority of cases, RNAi and mutants give concordant answers in identifying genes that are required to maintain muscle proteostasis. The RNAi experiments took roughly one-third to one-quarter of the time required for the genetic constructions, demonstrating a key strength of RNAi. Only three genes were identified by mutation alone, all from dominant gain-of-function alleles. This suggests that combined use of dominant alleles and RNAi offers a promising approach to epistasis experiments. Seventeen genes were identified by RNAi alone suggesting that RNAi may be a more sensitive tool. In one respect RNAi does indeed appear to be a more sensitive tool: acute RNAi treatment in adults can reveal negative regulators of degradation alone. Past work with this system has required use of temperature sensitive mutants to confirm a role in negative regulation of degradation [7], [8], [9], [10]. For example, we can only identify *twk-18* as a negative regulator of protein degradation because we were able to induce degradation in fully developed muscle by a temperature shift of adults carrying a dominant temperature-sensitive allele, whereas we identified 51 potential negative regulators of degradation using RNAi alone.

**Table 1:** Genes identified as affecting proteostasis by mutation versus RNAi.

gene name	allele	encodes	Proteostasis affected in mutant	Proteostasis affected in response to RNAi
*aex-5*	*sa23*	Kex2/subtilisin-like	Y	Y
*egl-30*	*ad805*dm	Gq-alpha	Y	Y
*unc-23*	*e324*	Chaperone	Y	Y
*unc-52*	*e669*	Basement membrane HSPG	Y	Y
	*e669su250*ts		Y	Y
*unc-112*	*r367*ts	Mitogen inducible	Y	Y
*twk-18*	*cn110*ts,dm	K channel	Y	N
*unc-43*	*n498*dm	CaM kinase	Y	N
*unc-105*	*n490*dm	Degenerin	Y	N
*egl-1*	*a487*	Cell death activator	N	Y
*egl-2*	*n693*dm	K channel	N	Y
*egl-5*	*n486*	TF	N	Y
	*n945*		N	Y
*egl-15*	*n484*	FGFR	N	Y
	*n1477*		N	Y
	*n1783*		N	Y
*egl-36*	*n728*dm	K Channel	N	Y
*egl-43*	*n1079*	Zn Finger	N	Y
*exp-3*	*n2372*dm	Calcium activated K channel	N	Y
*unc-5*	*e53*	Netrin Receptor	N	Y
*unc-24*	*e138*	Stomatin	N	Y
*unc-32*	*e189*	Vac H-ATPase	N	Y
*unc-36*	*e251*	Ca channel	N	Y
*unc-42*	*e270*	TF	N	Y
*unc-47*	*e307*	GABA transporter	N	Y
* *unc-51*	*e369*	Kinase	N	Y
*unc-103*	*e1597*dm	K channel	N	Y
*rol-6*	*su1006*dm	Collagen	N	Y
*sqt-1*	*sc13*	Collagen	N	Y

*unc-51 was identified as a potential false positive for RNAi inducing degradation.

### Assigning genes to functional groups regulating muscle protein degradation

**Figure 4:**
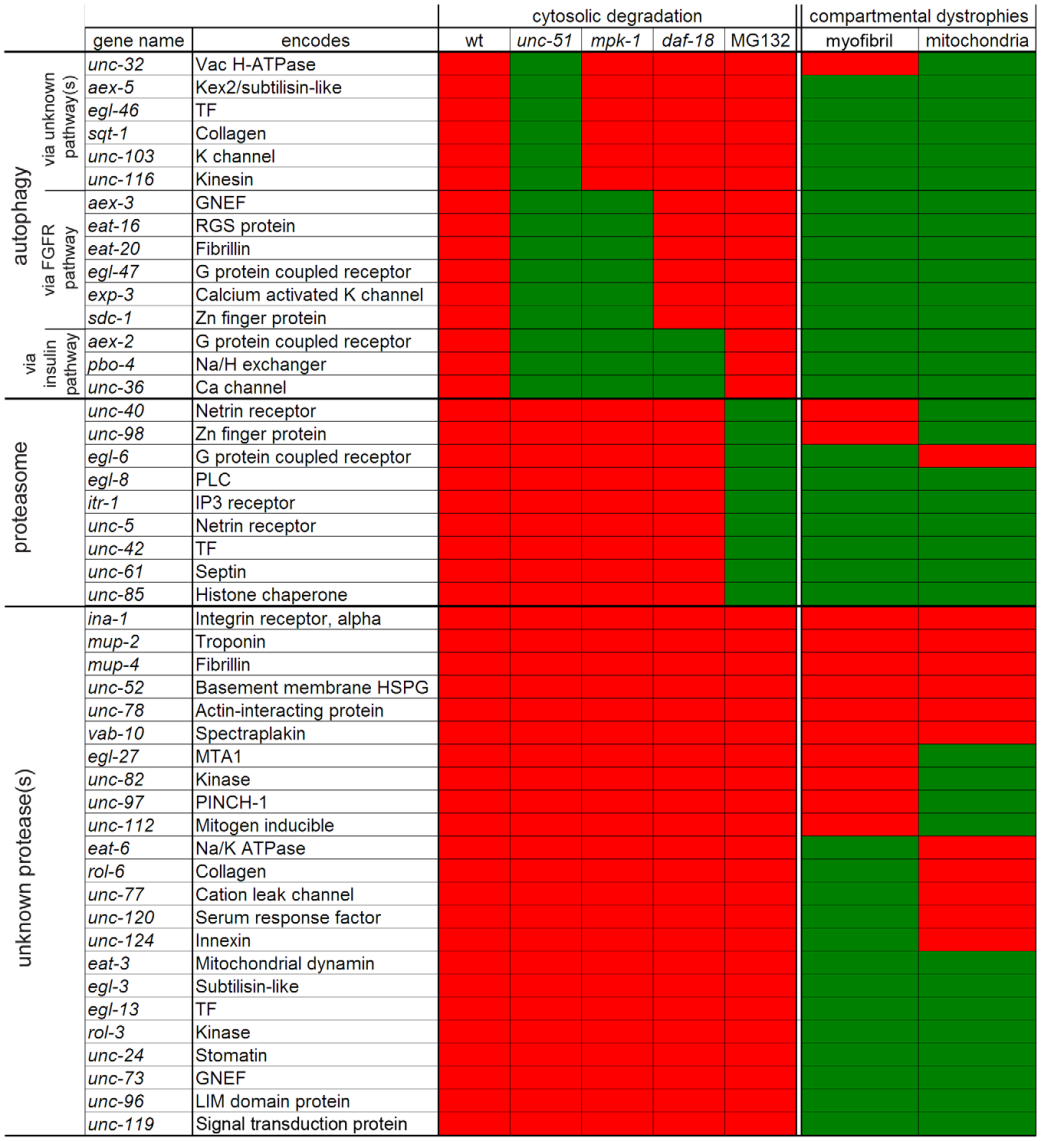
Identified genes negatively regulate muscle protein degradation via at least three functional clusters. Genes identified as negative regulators of protein degradation were examined for reproducibility (wt) and lack of degradation in autophagy pathway mutants (UNC-51 kinase (*unc-51*), Fibroblast Growth Factor pathway (*mpk-1*), Insulin like pathway (*daf-18*)) or when the proteasome was inhibited (MG132). A separate wild-type control was used in the autophagy and MG132 experiments. Displayed results indicate the result from two independent experiments or the consensus result from three. Degradation is denoted by red, lack of degradation by green. Results from other acute effects on muscle are listed on the right with dystrophies indicated in red and normal morphology indicated in green. Results are clustered based on putative degradation pathway: autophagy (top), proteasome (middle), unknown pathway(s) (bottom). The autophagy pathway is further subdivided as going through unknown pathway(s), going through the Fibroblast Growth Factor pathway but not Insulin like pathway, or going through both the Insulin like and Fibroblast Growth Factor pathways.

Having used RNAi to identify 51 genes whose products appear to negatively regulate bulk cytosolic protein degradation in *C. elegans* muscles, we next classified these genes into groups that act by common pathways or mechanisms. First, we used the data from Figure 2 to group genes according to which muscle compartment(s) were affected by acute RNAi treatment. Next, we conducted additional experiments to determine if the degradation we observed was related to the signalling pathways previously identified as negatively regulating cytosolic muscle protein degradation in *C. elegans* [7], [8], [9], [10]. The groups were established as follows: i) Lysosome-autophagy cluster: degradation blocked by *unc-51* reduction-of-function mutation [10]; MAPK signalling sub-cluster, degradation blocked by *mpk-1* reduction-of-function mutation [9]; IGFR signalling sub-cluster, degradation blocked by *daf-18* reduction-of-function mutation [10]; ii) Proteasome cluster: degradation blocked by proteasome inhibitor MG132 [7]. By this clustering methodology it appears that at least three distinct proteolytic mechanisms are regulated by the known muscle mutants (Fig. 4).

**Figure 5:**
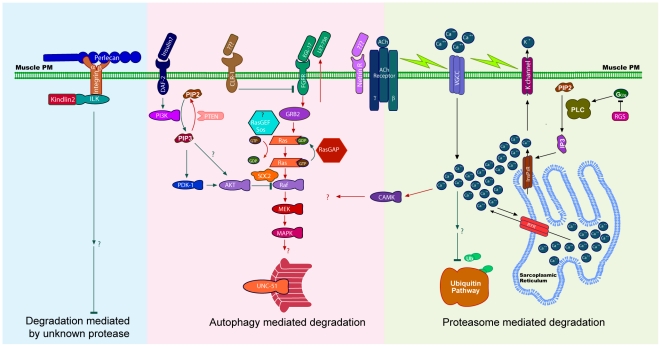
Preliminary model of control of degradation of bulk cytosolic protein in *C. elegans* muscle. Inferences from past [7], [8], [9], [10] and these studies are shown. Left (Blue): Degradation by an unidentified protease is negatively regulated by integrin based attachment complexes which bind to Perlecan in the extracellular matrix. Middle (Pink): Degradation by the lysosomes is controlled by a balance of signal from Insulin/Insulin-like Receptor (negative regulator, green lines) and autocrine Fibroblast Growth Factor signal (positive regulator, red lines). Calcium overload, signalling via CaMKII, also promotes lysosome-based degradation. Right (Green): Intra-cellular calcium controlled by a combination of membrane depolarization and G-protein signalling events is required to negatively regulate proteasome-based degradation. **N.B.** Genetic disruption of the pathway in the left section typically yields dystrophic myofibrils, whereas disruption of the pathways in the middle or on the right typically does not.

Consistent with past results, genes identified as negative regulators of lysosome or proteasome based degradation infrequently (4/24 genes) showed acute defects in gross myofibrillar or mitochondrial structure, suggesting that activation of bulk cytosolic degradation via lysosomes and proteasomes does not necessarily cause problems with other sub-cellular compartments. The lysosome-based cluster (15 genes) was completely non-overlapping with the proteasome-based cluster (9 genes). While this is in apparent contrast to the functional coupling between proteasome and autophagic pathways in non-muscle cells where inhibition of proteasome activity enhances autophagic activity [29], for overlap to occur in our analysis a gene would need to be a negative regulator of both the proteasome and autophagy (N.B. When the dominant gene data are included we do find functional coupling between the proteasome and autophagy: Calcium is a negative regulator of the proteasome and a positive regulator of autophagy (Fig. 5)). The partial overlap between the MAPK-based cluster and the lysosome-based cluster identifies 9 genes that may impinge on signaling upstream of MPK-1 MAP kinase [9] with 3 likely acting via IGFR based signaling upstream of Raf [10]. The 6 genes in the non-overlapping set may impinge on signaling downstream of (or in parallel to) MPK-1, yet upstream of UNC-51.

We also identified genes that do not appear to cluster with regulators of lysosomal or proteasomal degradation; this was the largest group of genes identified. These genes presumably regulate at least one additional protease although a more complex scenario is possible, such as dual regulation (with compensation) of proteasomes and lysosomes. However, given that knockdown of a gene in this group frequently affects the myofibrils and/or mitochondria (15/23 genes) it seems likely that calpains and/or caspases are being activated. The one new negative regulator identified by mutation alone, *twk-18*, appears to be in the proteasome cluster, since protein degradation is prevented by MG132.

Bioinformatic analysis, using WormBase [27], of genes in each of the groups suggests that there are specific regulatory networks awaiting confirmation via additional experiments. As examples: In the lysosome cluster, there are two Guanine nucleotide-binding (G) protein coupled receptor genes and a Regulator of G-protein Signalling (RGS) gene. In the proteasome cluster we found two netrin receptor genes, a phospholipase C (PLC) gene and an inositol triphosphate (IP3) receptor gene. In the third cluster are a large number of genes that are known to be involved in muscle attachment to the extracellular matrix and the Serum Response Factor (SRF) gene (a gene linked to muscle defects in humans [30]). A speculative model incorporating published data [7], [8], [9], [10] and our new dominant allele and acute RNAi data is shown in Figure 5.

In these additional experiments we were unable to further replicate results previously obtained for four genes (*mua-6*, *unc-51*, *dpy-2*, *pbo-6*), a potential false positive rate of 8% (N.B. these may be genes for which RNAi produces variable results). This reinforces our belief that appropriate replicates and controls can allow RNAi to be used very effectively in identifying genes that negatively regulate cellular processes with well-defined phenotypes.

## Discussion

We have used a combination of forward-genetic (mutational) and reverse-genetic (RNAi) approaches to interrogate a list of known genes for novel functions. Reassuringly, we have found that RNAi results are largely reproducible and match results from mutants. Key strengths of RNAi appear to be speed of use and the ability to acutely knock down gene function in fully differentiated adult muscle. However, a weakness of the RNAi approach appears to be the need for more stringent controls and/or replicates in order to achieve a high level of confidence that a gene has been correctly identified as relevant, before proceeding to more costly and time-consuming mechanistic studies. An important open question is whether more controlled and reproducible dsRNA dosing can be achieved by bacterial feeding, as this may reduce the variability of results obtained by RNAi. A combined approach utilizing mutants and RNAi should help offset these weaknesses, as previously suggested [18]. For example, once genes are identified and verified by RNAi and mutations, mutants can be used in combination with RNAi for epistasis experiments.

Based upon studies in mammals, we would anticipate a minimum of four regulatory subnetworks controlling protein degradation in *C. elegans* muscle: one each for the proteasome, lysosomes, calpains, and caspases. Previous work with *C. elegans* established at least one subnetwork for the ubiquitin-proteasome system [7] and the lysosomes [8], [9], [10]. Here we have identified additional genes that either fit within these existing subnetworks, or represent additional subnetworks controlling the same proteases. We have also identified a group of genes that represent at least one additional signalling system controlling at least one additional protease, presumably calpains and/or caspases. Current estimates suggest that there are from 1300 [31] to 8000 [32] genes that are enriched in *C. elegans* muscle. Similarly there are greater than 1000 genes for which RNAi yields an Unc phenotype, as indexed in WormBase [27]. Since the *unc* genes displayed the greatest variability in distribution of sub-cellular defects, it is possible that further examination of genes in this class will reveal more regulatory mechanisms than further examination of genes for which RNAi knockdown produces phenotypes associated with the other muscle mutant classes. Given that roughly a quarter of the *unc* genes we analysed displayed protein degradation upon knockdown, the regulation of degradation may be far more complex than initially suspected. However, it currently seems unlikely that these complex subnetworks regulate more than a handful of key proteases. Thus, it may be that despite large numbers of specific genes whose products impinge on these subnetworks, there are a limited number of general themes by which this network functions.

Fundamental metabolic pathways are largely conserved from single celled organisms to mammals [33] and the attachment and development of myofibrils is largely conserved between *C. elegans* and man [34]. Thus, it may be heuristically useful to conjecture that the general themes and some of the specific molecular mechanisms of regulation of muscle proteostasis are also largely conserved (even though it is already clear that the signal-transduction systems of *C. elegans* are considerably simpler). Nutritional status and use are well known to control human muscle size. However, we know surprisingly little of how this occurs at the molecular level. Our results point to some of the complex molecular signals that regulate muscle size in response to both activity and nutritional status. As altered contact with the extracellular matrix does not appear to impinge upon the key proteolytic systems (lysosomal, proteasomal), it may be that mechanotransduction *per se* [35] does not directly contribute to controlling degradation based muscle size changes in response to use even though attachment to the matrix is important for growth and maintenance/repair of muscle. This may suggest that earlier signals, in developmental terms, provide the framework upon which other, later signals can function to control muscle size. Calcium would appear to be a key player in terminally differentiated muscle, mediating the abilities to grow and shrink in response to use (it has also previously been identified as a regulator of proteostasis [36], [37], [38]). Factors that control depolarization of the muscle plasma membrane, including but not limited to contractile signal from nerves, control calcium release from the sarcoplasmic reticulum, which in turn negatively regulates proteasome-based protein degradation. It is an open question whether there are specific targets of calcium, and/or if calcium release from the sarcoplasmic reticulum (in)directly affects proteostasis [38]. Conversely, calcium overload triggers protein degradation via autophagy, which also is controlled by growth factors. Given that growth factor signalling can control both autophagy and protein synthesis via mTOR [39], one attractive model is that CaMKII coordinates calcium overload signal upstream of TOR, thereby allowing muscle to directly integrate control of muscle proteostasis in response to both nutritional status and activity. This model also suggests that there is a key temporal element to maintenance of muscle size, since calcium overload, and consequent protein degradation, are likely to be transient. This is consistent with the observation in humans that protein degradation goes up transiently following exercise [40]. Thus, our data suggest that further mechanistic work is required in *C. elegans*, and that even if identical signals are not acting in human muscle, orthologous ones are likely to be important. More broadly, as calcium [41], and other [42], channels can be degraded, in non-muscle cells by the proteasome to prevent trafficking to the plasma membrane and can also be degraded by the endosome-lysosome system once at the plasma membrane, our results raise the question of whether we have uncovered a fundamental, feedback controlled, mechanism of control of proteostasis: plasma membrane polarization.

## Materials and Methods

### Nematode handling and genetics

Nematode strains were maintained and grown at 20°C as described [23]; temperature sensitive mutants were routinely maintained at 16°C. All alleles used in this work are listed in Tables 1 and S2. The transgene used for assessing muscle-specific proteostasis and protein degradation was *ccIs55* (*unc-54::lacZ*) with histochemical staining for LacZ activity as described [23]. The transgene used for assessing the myofibrils was *jIs01* (*myo-3::GFP*), a translational fusion of the full-length *myo-3* (myosin heavy chain A) gene to GFP, with epifluorescence microscopy as described [22]. The transgene used for assessing the mitochondria and nuclei was *ccIs4251* (*Pmyo-3::MitGFP; Pmyo-3::NLS::GFP-lacZ*) with epifluorescence microscopy as described [17]. Many strains containing one of these transgenes and mutant alleles of “muscle genes” were constructed specifically for this work, while others were earlier constructed in the Jacobson lab. All strains were constructed using standard techniques [12]. Full details of constructions and strain genotypes are available upon request.

### RNAi screening of behavioural, developmental and sub-cellular phenotypes

RNAi using bacterial feeding vectors was performed essentially as described [19], [20] using PD55 (*ccIs55* V), PJ727 (*jIs01*; *ccIs55* V), and CB5600 (*ccIs4251* I; *him-8*(*e1489*) IV). RNAi feeding vector clones used in these studies are listed in Table S1. Chronic RNAi exposure experiments were conducted by placing 3–4 L4-stage hermaphrodite worms onto NGM RNAi plates containing seeded bacteria expressing dsRNA for each gene and then incubating for 72–96 hours at 20°C. Progeny were then scored for developmental phenotypes with young adults scored for muscle dystrophies and cytosolic protein levels by LacZ staining. An additional examination of muscle dystrophies and cytosolic protein levels were made in adults again 24 hours later. L4 hermaphrodites from the F1 generation were then transferred to newly seeded NGM RNAi plates with the same bacteria for examination of the F2 generation as above. Developmental/behavioural phenotypes scored were: Unc (uncoordinated movement), Rol (rolling movement), Bmd (abnormal body morphology), Dpy (short fat appearance), Pvl (protrusion from the vulva), Rup (rupture from the vulva), Ste (sterile), Egl (egg laying defective), and Gro (long period of development and/or growth arrest). Developmental phenotypes were recorded if >10% of worms on the plate showed a phenotype and also if the same phenotype was observed in both generations, even in fewer than 10% of total worms. Sub-cellular phenotypes scored were: Cytosolic protein content (normal, abnormal), myofibrillar morphology (normal, abnormal), mitochondrial morphology (normal, abnormal); see Fig. 1 for examples of normal and abnormal. In all cases abnormalities deemed minor (e.g. not appreciably different from vector control) were scored normal and abnormal if moderate and/or severe (in comparison to the vector control) in at least 20% of worms on the slide. Defects, within an individual worm, were classed as moderate as follows: i) cytosolic protein content: at least a 30% loss of stain (e.g. intensity); ii) myofibrillar morphology: at least 2 disorganized or broken myofibrils in at least two muscles; iii) mitochondrial morphology: loss of at least 25% of the mitochondrial network in at least two muscles. Defects, within an individual worm, were classed as major as follows: i) cytosolic protein content: at least a 90% loss of stain (e.g. intensity); ii) myofibrillar morphology: at least a 90% lack of recognizable myofibrils in at least two muscles; iii) mitochondrial morphology: at least an 80% lack of networked mitochondria network in at least two muscles. Genes that were identified as negatively regulating muscle dystrophy or cytosolic protein content in chronically treated animals were then examined for effects of acute RNAi exposure. For acute RNAi exposure, worms were roughly age synchronized [23] and grown to young adulthood on non-RNAi bacteria at 20°C (approximately 48 hours). Young adult animals were manually transferred to NGM RNAi plates and maintained at 20°C. A group of adults was examined for muscle dystrophies and cytosolic protein degradation at adulthood and 24, 48, and 72 hours post adulthood. Dystrophies and degradation were scored as positive if they appeared at any two time points after introduction to RNAi, with particular attention to progressive loss of cytosolic protein between the 48 and 72 hour time points. Comparisons with previously published phenotypes using the same RNAi clone were made using WormBase (phenotypes: RNAi details).

### Clustering genes into pathways

Mutants and drugs for clustering identified genes were as described [7], [8], [9], [10]. Acute RNAi experiments as described above were conducted in wild-type, mutant and drug treated worms carrying appropriate transgenes. Degradation was scored as above in two independent experiments; in case of discrepancy a third experiment was run.

## Supporting Information

Figure S1 **Reproducibility of developmental and/or behavioural phenotypes produced by** ***C. elegans*** **RNAi feeding vectors.** A) Analysis of overall ability to observe a consistent phenotype in our study vs. past studies using the same feeding vectors [19], [20], [21]. The total number of genes examined in this study was 159. Fractional analysis uses number of genes on the left or percentage of 159 genes on the right (top line). Observations were scored as consistent if any of the past reported phenotypes were observed in this study. This analysis reveals a general agreement but with a substantial number of inconsistencies between this study and past studies. B) Analysis of the nature of consistent developmental/behavioural phenotypes reported in this and past studies. This analysis reveals the majority of consistent phenotypic observations were the lack of a visible phenotype. C) Analysis of the nature of inconsistent phenotypes reported in this and past studies. Observations were scored as inconsistent if none of the past reported phenotypes were observed in this study. This analysis reveals that the majority of genes for which our observations are not consistent with past observations using these same clones we actually observed a phenotype and this observed phenotype was consistent with the known mutant phenotype. This analysis also reveals an inability to find convergence in phenotypes produced 6% of the time and a known false negative rate of 3%. D) Analysis of if RNAi clones that do not produce a developmental/behavioural phenotype do produce a sub-cellular defect in muscle. This analysis reveals that the majority of genes for which RNAi treatment does not produce a phenotype (Figures from the last lines of B and C are considered together), RNAi treatment does produce a sub-cellular defect.
(TIF)

Figure S2 **Genes that do not affect muscle protein synthesis, degradation and/or dystrophy.** Effect of RNAi on cytosolic proteostasis, degradation, myofibrillar development, myofibril maintenance, mitochondrial development, and/or mitochondrial maintenance was not observed for these genes. Each gene is named to the left. Genes are broken into classes: A) *eat* mutants; B) *aex*, *exp*, *pbo*, *dec* mutants; C) *mua*, *mup*, *rol* mutants; D) *egl* mutants; E) *unc* mutants. Genes within each class are clustered by whether an organismal level phenotype was observed (yellow) or not (white). Examples of sub-cellular phenotypes that were scored are provided in Figure 1 and genes from the same classes that displayed sub-cellular defects are shown in Figure 2.
(TIF)

Table S1 **Comparison of developmental and behavioural phenotypes observed with those previously reported.** All genes for which RNAi was used to study the effects of decreased gene expression are listed. Alternative names for the genes, where indexed in WormBase, are listed. The RNAi clone used in this study is listed. Clones starting with a Roman numeral are from the MRC library while clones starting with an Arabic number are from the Open Biosystems library. A brief summary of what the gene is thought or known to encode is provided based upon the information in WormBase. Developmental and/or behavioural phenotypes scored by us or others are listed. Details of phenotypes scored by us can be found in Material and methods. Details of phenotypes scored by others can be found in the indicated references. For comparison the known mutant phenotype, as indexed in WormBase is provided.
(XLS)

Table S2 **Genes not identified as affecting proteostasis by mutation versus RNAi.** All genes for which we failed to detect decreased protein synthesis or increased protein degradation, by decreased level of transgenic reporter protein (see Materials and methods), are listed. Specific alleles tested are indicated. Five genes, where dominant alleles existed, were tested by mutation alone. This table is a companion to Table 1, in the main text, which displays all genes for which we did detect decreased protein synthesis and/or increased protein degradation.
(XLS)
