# Supplementary figures and images for: Identification and Functional Clustering of Genes Regulating Muscle Protein Degradation from amongst the Known C. elegans Muscle Mutants
Source: PLoS One. 2011 Sep 27;6(9):e24686. doi: 10.1371/journal.pone.0024686 (PMC3181249; doi:10.1371/journal.pone.0024686)

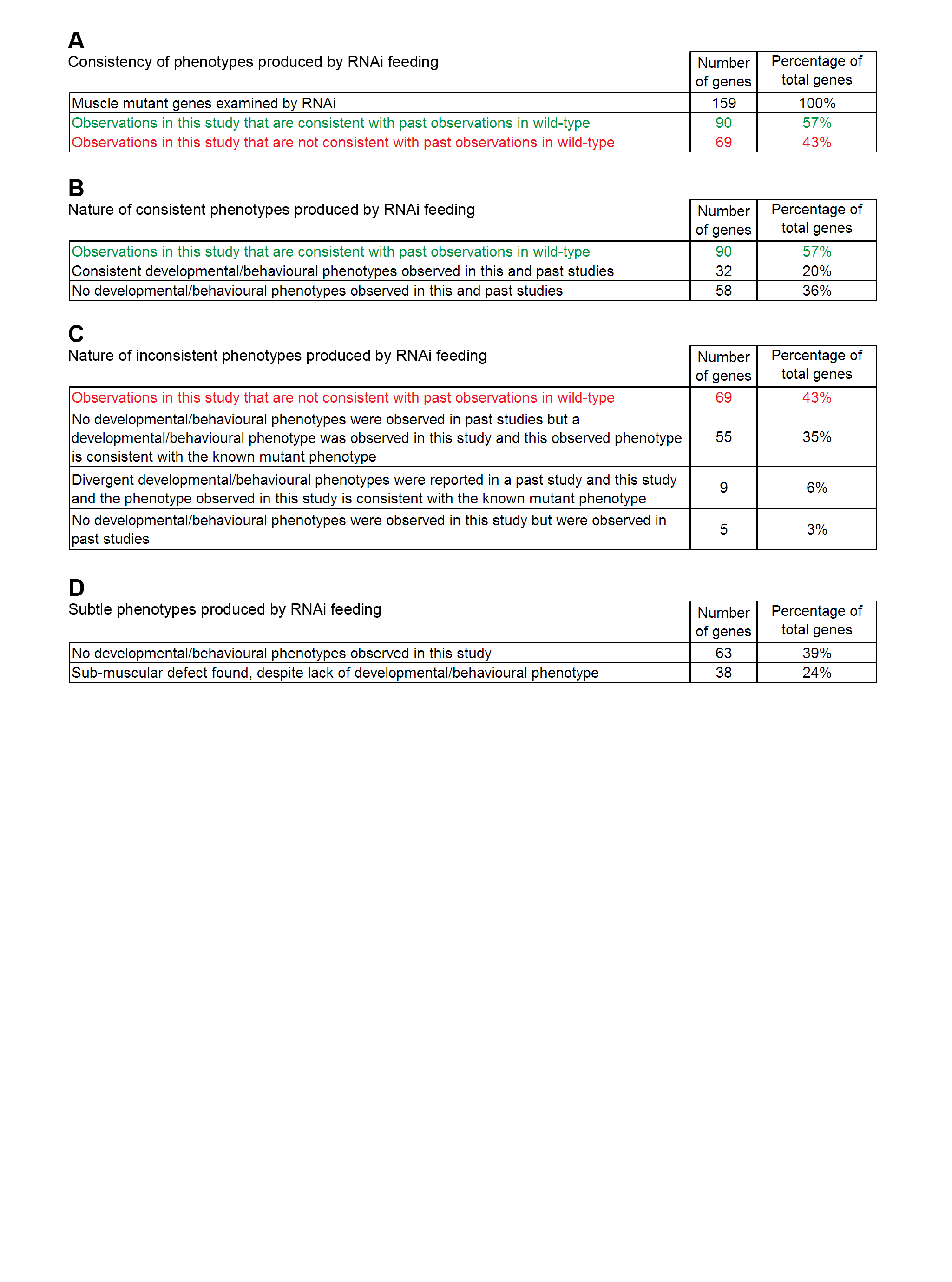

Supplement: Figure S1 — Reproducibility of developmental and/or behavioural phenotypes produced by C. elegans RNAi feeding vectors. A) Analysis of overall ability to observe a consistent phenotype in our study vs. past studies using the same feeding vectors [19], [20], [21]. The total number of genes examined in this study was 159. Fractional analysis uses number of genes on the left or percentage of 159 genes on the right (top line). Observations were scored as consistent if any of the past reported phenotypes were observed in this study. This analysis reveals a general agreement but with a substantial number of inconsistencies between this study and past studies. B) Analysis of the nature of consistent developmental/behavioural phenotypes reported in this and past studies. This analysis reveals the majority of consistent phenotypic observations were the lack of a visible phenotype. C) Analysis of the nature of inconsistent phenotypes reported in this and past studies. Observations were scored as inconsistent if none of the past reported phenotypes were observed in this study. This analysis reveals that the majority of genes for which our observations are not consistent with past observations using these same clones we actually observed a phenotype and this observed phenotype was consistent with the known mutant phenotype. This analysis also reveals an inability to find convergence in phenotypes produced 6% of the time and a known false negative rate of 3%. D) Analysis of if RNAi clones that do not produce a developmental/behavioural phenotype do produce a sub-cellular defect in muscle. This analysis reveals that the majority of genes for which RNAi treatment does not produce a phenotype (Figures from the last lines of B and C are considered together), RNAi treatment does produce a sub-cellular defect. (TIF) [file pone.0024686.s001.tif]

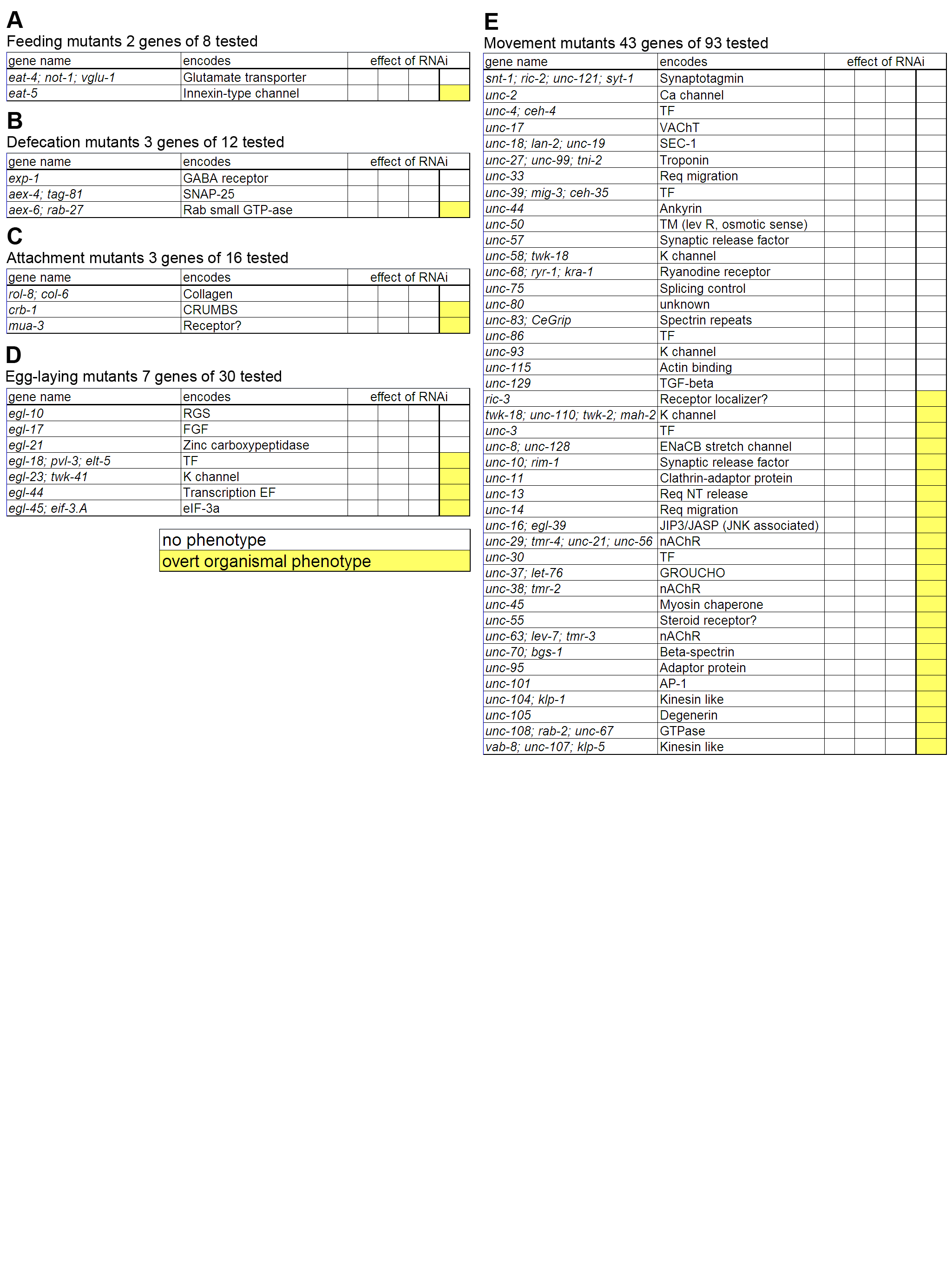

Supplement: Figure S2 — Genes that do not affect muscle protein synthesis, degradation and/or dystrophy. Effect of RNAi on cytosolic proteostasis, degradation, myofibrillar development, myofibril maintenance, mitochondrial development, and/or mitochondrial maintenance was not observed for these genes. Each gene is named to the left. Genes are broken into classes: A) eat mutants; B) aex, exp, pbo, dec mutants; C) mua, mup, rol mutants; D) egl mutants; E) unc mutants. Genes within each class are clustered by whether an organismal level phenotype was observed (yellow) or not (white). Examples of sub-cellular phenotypes that were scored are provided in Figure 1 and genes from the same classes that displayed sub-cellular defects are shown in Figure 2. (TIF) [file pone.0024686.s002.tif]
